# The Significance and Treatment of Uterine Hemorrhage

**Published:** 1908-04

**Authors:** William Edwards Fitch

**Affiliations:** Lecturer on Surgery, Fordham University School of Medicine; Attending Gynecologist Presbyterian Hospital Dispensary; Attending Physician Vanderbilt Clinic College of Physicians and Surgeons, New York City


BUFFALO MEDICAL JOURNAL.

VOL. LXIII. APRIL, 1908. No. 9

### ORIGINAL COMMUNICATIONS.

### The Significance and Treatment of Uterine Hemorrhage

BY WILLIAM EDWARDS FITCH, M. D.

Lecturer on Surgery, Fordham University School of Medicine; Attending Gynecologist Presbyterian Hospital Dispensary; Attending Physician Vanderbilt Clinic College of Physicians and Surgeons, New York City.

THE history of gynecology is accentuated by progress. In glancing over its pages the student is astonished at the rapid strides made to alleviate the ills of womankind. It is a chapter written within the present century, and written with glowing pen and on emblazoned paper, recording the daring of Ephraim McDowell, of Kentucky, who in 1809 performed the first laparatomy on a poor woman from the wilds of the blue grass state, without anesthesia, an event which marked the beginning of intrapelvic gynecologic surgery.

Perhaps the brightest page in this chapter contains the wonderful discovery of Crawford W. Long of Georgia, who on March 30, 1842, used ether for the first time as an anesthetic, while operating on James Venable, from whose neck he removed without pain a large tumor—robbing, for all time, the operating table of its dreadful horrors. Another page in this chapter records the brilliant labors of Marion Sims, a native of South Carolina, who in 1845-9 described for the first time his operation for vesico-vaginal fistula." He also gave to the profession the duck-bill speculum which bears his name, for the exposure of the fistula with the patient lying in the left semiprone position. Elsewhere in this same chapter we find a description of Battey's operation first performed by Robert Battey, of Rome, Ga., who on August 17, 1872, performed the first ophorectomy in this country upon a nervous, hysterical woman whose menstrual periods, prior to the operation, almost drove her to madness. This operation was successful, adding years of joy and happiness to his former invalid patient.

New operations, improved surgical technic, and new therapeutic remedies have been the means of giving the suffering woman a new lease on life. These new methods and measures must usurp the attention and command the coöperation of every thoughtful gynecologist. He must forsake his ultra conservatism and pay due homage to whatever in his experience has most abundantly redounded to the amelioration of the ills of suffering woman.

The gynecologist should be a good listener, that he may fully grasp the importance of the symptoms which his patient confidingly recites. He must be able to judge human nature in order to carefully weigh the evidence submitted and correctly interpret its bearing on the case. When a woman comes for consultation and gives a history of uterine hemorrhage, we should be very thorough in our interrogatory examination, since bleeding from the uterus is one of the most common complaints for which the physician is consulted. The causes are numerous and the diseased conditions manifold.

This paper is intended to be intensely practical and the writer hopes his labors may be of some help to his fellow practitioners, and thereby benefit womankind. If we succeed we shall feel amply repaid for the time and labor spent in its preparation. In considering the treatment of uterine hemorrhage, we have before us a subject of momentous importance and one requiring much thoughtful study. Hemorrhage from the uterus may indicate:

*Adenoma Malignum*—Here the hemorrhage is less profuse, the discharge is less abundant, and the odor less offensive than in cancer.

*Cancer of the Body of the Uterus*—Here the bleeding may be intermittent and copious or constant and dribbling.

*Cancer of the Cervix*—A persistent watery discharge tinged with blood, usually associated with fetor; later, profuse and violent hemorrhage may occur.

*Deciduoma Malignum*—In this condition there is intermittent hemorrhage, usually severe, followed by an offensive, turbid watery discharge,—bloodclots may be found.

*Ectopic Gestation*—Agonising pain, faintness and collapse are indications that the tube has ruptured and violent hemorrhage present.

*Endometritis*—This begins with a dull, aching pain accompanied by purulent and oftentimes bloody secretion, menorrhagia and metrorrhagia being common.

*Fibroid Polypi*—Here will be found a profuse, purulent discharge; menorrhagia with colicky or propulsive pains, the bleeding dribbling or severe flooding.

*Fibroid Sarcoma*—In this there may be a torrential hemorrhage occurring with alarming suddenness and severity, or there may be only a continual dribbling as in cancer of the body of the uterus.

*Inversion of the Uterus*—Here the hemorrhage is very profuse accompanied by a dragging or pulling sensation.

*Lacerated Cervix*—In this condition the hemorrhage is usually slight.

Ophoritis (chronic)—This condition is attended by menorrhagia, especially in cystic ovary, which gives rise to a most intractable form of uterine hemorrhage.

*Ovarian Cyst*—In this as in cystic ophoritis, menorrhagia and metrorrhagia are common.

*Retrodisplacements*—Menstruation usually profuse with aggravated reflex disturbance in these conditions.

*Salpingitis*—In this there is increased frequency and duration of menstruation, bloody discharge accompanied by pain, quick pulse and persistent elevation of temperature.

*Simple Cysts of Ovary*—Here, too, there is occasional hemorrhage and metorrhagia.

*Subinvolution of the Uterus*—In this condition the symptoms are backache, anemia and malaise, depression of spirits, profuse menorrhagia.

*Uterine Fibroids*—In these the symptoms usually are pressure, pain and hemorrhage either at or in the intervals of menstruation.

*Uterus, prolapsus of*—There is pelvic pain, with weight and dragging sensation in acute prolapse, then, when the ligaments are torn, profound shock and agonising pain attended with internal hemorrhage.

The above are a few of the diseased conditions that we will be called upon to treat which will be accompanied by uterine hemorrhage. Some of these cases will be easily diagnosticated and yield readily to treatment while others will come to us in which no abnormal signs can be found on physical examination, and in which treatment will prove extremely unsatisfactory. Still there are other cases which can be cured only by surgical intervention. Many of the patients presenting themselves for consultation will be women between twenty-five and thirty-five years of age,—a period of life when menstruation should be regular and normal. In many of these sufferers, beyond a slight enlargement of the uterus and a somewhat toneless condition of the uterine muscle and appendages, there is nothing abnormal which can be discovered on the most painstakingly careful bimanual examination. While operative gynecology has made rapid advancement saving the lives of many women, the non-surgical conservative workers have been "up and doing." Scientific pharmacologists and chemical laboratory workers have greatly aided the conservative gynecologists.

Whatever may be the correct interpretation of the pathological changes present in uterine hemorrhage, the fact remains that the control of the bleeding is often a very difficult problem, especially in those cases unsuitable for surgical intervention, and the drugs which we have at our command for the control of hemorrhage often prove disappointingly unsatisfactory. Frequently cases come under observation in which the usual hemostatics and styptics, such as ergot, hydrastis, hammemelis, gallic and tanic acids, persulphate of iron and other drugs of this type have been used without even the slightest signs of alleviating the hemorrhagic flood. The cervix has been dilated, the endometrium cureted and still the patient bleeds. In the past, in such cases, the woman had to give up her organs or bleed to death. The writer recalls a patient who literally bled to death, ten years ago, because she refused hysterosalpingoöphorectomy—the only chance of saving her life.

Conservatism in gynecology has been a stimulus to the broadening of therapeutic effort and has saved countless numbers of women from needless sacrifice of organs and other surgical mutilations that in so many instances failed to bring relief. We are sometimes compelled to remove a uterus because of our inability to check uterine hemorrhage permanently, as its recurrence is often to such an extent as to greatly endanger the life of the patient, and when the organ has been extirpated the pathologist will open his eyes in surprise to see how comparatively trifling the organic changes will be. If uterine hemorrhage occurs after parturition or abortion, we know the cause—faulty contraction of uterine muscular fiber. The open bloodvessels gape through the atony of the muscle and we all know that any remedy which causes firm contractions of the uterine muscle will stop hemorrhage. A uterus infiltrated by small fibromyomata, or which is chronically inflamed, is an entirely different case. In such cases an abnormal growth of tissue is present, a growth which compresses the muscular fibers, hindering them from contracting with sufficient force to control arteriosclerotic ones which gape wide open in spite of muscular contractions, due to the rigid surrounding connective tissue.

In these cases, only such remedies are worth the while, except those which act independently of muscular contraction. Dr. Abel of Berlin[1] says he has had occasion to examine microscopically uteri which had been removed on account of uterine hemorrhage when all other treatment had failed. He avers on examining these uteri he has observed that the capillaries are greatly dilated in the múcous membranes which have been the seat of severe hemorrhage, therefore he declares that ulcerative processes were present exposing the vascular loops on the surface of the uterine mucous membranes.

We can readily understand how such uteri become the seat of extensive hemorrhage and that this bleeding can only be controlled by a remedy which influences the vasomotor nervous system directly, promptly counteracting the irritation that induces the dilatation of the capillary vessels. Such a remedy is the neutral phthalic acid salt of cotarnin.

Cotarnin was discovered by Woehler[2] who obtained it by the oxidation of the opium alkaloid, narcotine, by means of manganese dioxide and sulphuric acid.

*Pharmacology*—Cotarnin phthalate is a yellowish microcrystaline powder containing 75 per cent. of cotarnin. It is readily soluble in water with a feeble alkaline reaction. Its melting point is 113° C and it is represented by the formula—$(C_{12} H_{16} NO_3)_2 C_3 H_6 O_4$. It contains two active hemostatics, as not only the cotarnin is noted for its hemostatic and sedative action, but valuable styptic properties are also claimed for the phthalic acid. According to Lockyer[3] cotarnin is chemically related to hydrastine, the latter containing stypticin, the hydrochloride of cotarnin.

*Physiological Action.*—Cotarnin phthalate exerts no action on normal bloodvessels, nor does it produce a rise of blood pressure, but its action in the main is directed to the capillaries dilated by inflammatory changes. Its physiological action on the uterus has been studied by Mohr[4] and Abel of Berlin and by Chiappe and Ravano in Boss's Clinic at Genoa[5]. These investigators agree that cotarnin displays not only a powerful hemostatic, but also a sedative action. It might be supposed that as a derivative of opium it would depend for its hemostatic action upon central causes, but Vieth and others aver that this is not so, since the drug does not occasion a general rise in blood pressure: he regards the uterine hemostasis as a purely local action, and supports this view by the statement that when used externally it causes hemostasis by vasoconstriction.

Quoting from Lockyer he believes that after absorption by the blood "cotarnin has the peculiar property of causing constriction of the urogenital vessels only, this action he avers is caused by the stimulation of their local vasomotor plexuses. Cotarnin does not effect normal vessels in other parts of the organism, hence a general rise in blood pressure does not occur. While its action is very prompt in the arrest and control of uterine hemorrhage yet it does not allay the bleeding in hemoptysis and hematamosis.

*Toxic Effects.*—After the administration of a lethal dose says Abel and Mohr, there first appears a sedative effect, exactly as with small doses, but this is soon followed by excitation, which is preceded by incoördination and ataxia of the extremities. This excitation is rapidly followed by respiratory and general paralysis and death. Just before death the number of respirations rapidly decrease and the breathing gradually becomes superficial. To produce poisonous effects very large doses must be given and Mohr has proven that gangrene cannot be set up by the continuous exhibition of this drug.

*Therapy.*—Mohr's experiments on pregnant rabbits establishes the fact that the uterus is rendered less sensitive to stimulation by the sedative action of cotarnin. This reduction in the excitability of the uterine nerves is an indication for its employment for the relief of spasmodic and congestive dysmenorrhea, and from the mass of clinical evidence collected by competent clinicians it establishes the opinion that in it we have a drug which fulfills a unique position in therapeutics, and one which can be safely given in threatened abortion. If the deductions of the German clinicians as to the physiological action of cotarnin are correct, and we believe they are, then in this drug we have a substance which has a special selective action upon the uterine nerve plexuses, which action produces a local vasoconstriction, at the same time, from its sedative action, pain is relieved. As it causes no contraction of uterine muscular tissue it is not indicated for the relief of post-partum hemorrhage, nor for any pathological condition where it is necessary to secure continuous retraction of uterine muscle.

The first clinical research into the therapeutic application of this drug was carried out by Katz[6] in Professor Karl Abel's clinic in Berlin. He and Abel enunciated the following deductions, dose 3 to 5 grains daily in original sugar-coated tablets, and the following indications for its use were laid down. After an experience of several years, in more than 300 cases, Katz and Abel recommend it in the following cases, namely:

1. Severe menstrual hemorrhage in virgins and nulliparæ without a pathological-anatomical cause.

2. In purely climacteric hemorrhage.

3. In puerperal hemorrhage.

4. In myomal hemorrhage, especially in cases suitable for operations; the patients, generally enfeebled by the recurring attacks of menorrhagia are âble to recover their strength when the bleeding is held in check by cotarnin phthalate.

5. In secondary hemorrhage in diseases of the appendages, or of the pelvic cellular tissue. (These are the cases where success is least certain, for many cases of this type are amenable to no other treatment than the removal of the actual cause of hemorrhage by surgical intervention).

6. In hemorrhage due to inoperable carcinoma, in which the local application of the remedy has also been used.

7. In dysmenorrhea, when not due to mechanical causes. Because of its double sedative and hemostatic action, this drug is a safe remedy for painful and prolonged menstruation. The sedative action is hardly ever absent, provided the remedy be given in sufficient doses,—2 tablets three to four times a day.

8. In hematuria,—bleeding from the genitourinary tract.

Abel in closing his paper, published in *Berliner Klinische Wochenschrift,* 1905, No. 34, summarises as follows: "I believe that in the neutral phthalate of cotarnin, (styptol[1]), we have gained a preparation which surpasses all former hemostatics employed in gynecology, provided the indications and dosage are right, whether administered internally, locally, or by a combination of both methods. Here I should like to mention once more that the remedy possesses a pronounced sedative effect which makes it especially valuable in gynecological practice." He quotes Freudenberg who is of the opinion that styptol cannot always replace ergotin; on the other hand, Professor Toff[8] observes that ergotin, and powdered ergot, have often proved unsuccessful, while styptol has always shown a reliable hemostatic effect. He even believes the time is not far distant when ergot preparations will lose their reputation just as they have long ago been given up as abortifacients for which they were used for decades. Weissbart[9] praises the action of the drug in climacteric and reflex hemorrhages, endometritis, subinvolution of the womb after parturition and miscarriage, and hemorrhages during pregnancy. Styptol never causes uterine contraction nor labor pains, an observation made by Freudenberg[10] which is in perfect accord of other German authors.

More than two years ago my attention was first drawn to this drug, as a remedy for the control of uterine hemorrhage. During that time I had occasion to observe its therapeutic action both in private and dispensary practice. My service, in one of the largest clinics for the diseases of women in the city of New York, has given me exceptional advantages for the study of this drug. I believe, as is claimed by the German clinicians, that the special action of the drug is principally on the capillary circulation, and that it has the power of contracting these dilated bloodvessels, in this way cutting short local congestion.

I have clinically tested the therapeutic properties of cotarnin phthalate for the arrest and control of uterine hemorrhage, and append hereto a few clinical histories taken at random from my note-book which will serve to illustrate the value of this drug in the treatment of uterine hemorrhage.

---

1. Styptol has been approved by the council of Chemistry and Pharmacy of the A. M. A.

In December 1905, I was called in consultation to see Mrs. S., aged 32, she first menstruated in her fourteenth year, was always regular as a girl, but suffered a great deal of pain during the periods, which were very profuse, married twelve years, two children and two miscarriages,—the last, two years ago; since then, her periods have been painful and alarmingly profuse, lasting sometimes from six to ten days, passing clots and suffering from frequent micturition; bowels constipated. Vaginal examination revealed a large and bulky uterus, slightly anteflexed, cervical catarrh, nil in fornices: diagnosis, endometritis with metorrhagia. Curettage advised but refused; patient was then placed on cotarnin phthalate, one tablet three times daily and advised to continue it until the next period, which she did, and after three months she reported freedom from her former ills.

Millis S., aged 31, menstruated first in her twelfth year; regular for the first two years, then cam[illegible] a period of 18 months during which she was very irregular. Acute ovarian pain preceding each period, beginning five or six days before and continuing for the first three days of the flow, which was very profuse and weakening to the patient. This case was first observed in February, 1906. Bowels constipated, micturation normal, appetite peevish, sleep disturbed. Vaginal examination revealed a slightly enlarged uterus freely movable but tender bimanually, some cervicitis with a slight erosion. Diagnosis, dysmenorrhea with menorrhagia. A curettage was performed by her physician during the early part of the preceding month. I was called again in April, same year, and found patient suffering with a return of her former symptoms. She was displeased at her condition and bewailed her plight after undergoing an operation which had been promised as a relief to her sufferings. She was placed upon neutral phthalate of cotarnin,—one tablet four times daily,—which was continued right along through the next period and until the next succeeding catamenia, both of which she passed through without any trouble or inconvenience.

Rebecca S., colored, aged 30 years menstruated first at twelfth year, regular for several years following; married three years, no children, no miscarriages. When I first saw her in April, 1906, she was complaining of headache, pain in her back, leucorrhea very profuse during intervals between periods, vaginal examination revealed uterus anteflexed, the sound glided in two and one-half to three inches, tip turning to the front. On bimanual examination a small mass was made out between the folds of the broad ligament on the left side; this mass was diagnosticated as a fibroid about the size of a small tangarine orange. Knowing the predisposition of her race to fibroids, she was placed on a tonic treatment and kept under observation. In July, same year, the second examination found the tumor much larger, firmly fixed laterally, but slightly movable in the vertical; acute pain in the left illiac fossa gave her considerable annoyance; she suffered nausea and vomiting during these attacks of ovarian pain; menses very profuse, occurring irregularly at periods varying from two to six weeks and lasting from six to eleven days. She was at once placed upon tablets of cotarnin phthalate, receiving four a day and after four months' treatment pain was very slight; menses about normal; size of tumor, slightly diminished; patient better.

Mrs. K., American, aged 38, menstruated first in her seventeenth year, usually regular, married twenty years, ten children and eight miscarriages,—the last miscarriage eight months previous—since which time she has suffered from a continuous metorrhagia: bowels constipated, appetite fickle, sleep disturbed, micturation frequent and irritating. In March, 1907, I examined this patient vaginally and found the cervix lacerated, hardened and enlarged, uterus enlarged circumferentially, left ovary prolapsed and very tender, left tube a boggy mass. Diagnosis salpingo-ophoritis; operation advised but refused; bleeding ceased after the administration of the neutral phthalate of cotarnin, one tablet every six hours for a period of four weeks. Of course in this instance the bleeding was controlled, but the diseased condition remains uncured.

Fannie R., German, aged 34 years, menstruated first in her fifteenth year, always regular, married thirteen years, two children, and six abortions; bowels constipated, appetite poor, sleep normal, micturation normal, complained of severe backache, pain over ovarian region, a bloating of lower abdomen and a continuous loss of blood since last abortion. On vaginal examination found vagina relaxed and enlarged, uterus mobile, os soft and gaping, ovaries tender. Diagnosis, subinvolution with metritis. Ordered woman to bed and turpentine stupe to abdomen, hot sterile douches at bed time and cotarnin tablets, one every six hours. After two weeks the bleeding began to subside and at the end of one month the loss ceased and did not recur.

These few examples of a large series of cases extending over two years of practice, suffice to show the value of cotarnin phthalate (styptol) to be a most favorable one in bleeding from the uterus. I have prescribed the drug chiefly in the disorders of menstruation, including dysmenorrhea, menorrhagia, and metorrhagia, metritis and endometritis and other congestive lesions of the womb. The experience of others, as well as myself, proves that in this drug the gynecologist has a reliable remedy for the control of uterine hemorrhage.

BIBLIOGRAPHY.

1. Abel—"On the administration of styptol in uterine hemorrhage and in dysmenorrhea." *Berliner Klinische Wochenschrift*, 1905. No. 34.

2. Woehler—*Annalen der Chemic and Pharmacie*. Vol. 1.

3. Lockyer—"Styptol in uterine hemorrhage." *Annals of Gynecology and Pediatry*, September, 1907.

4. Mohr—"Experimental researches on the effect of some uterine hemostatics, with especial reference to styptol." *Therapie der Gegen wart*, 1905, No. 16.

5. Chiappe and Ravano—"Styptol a new remedy in gynecological and obstetrical practice." *Archivo Italiano di Ginecologie*, 1904. No. 2.

6. Katz—"Styptol, a new remedy for uterine hemorrhage," *Therapeutische Monatshefte*, June, 1903 and November, 1904.

7. Freudenberg—"On Styptol." *Der Frauenarzt*. No. 3. 1904.

8. Toff—"Styptol, or Stypticine." *Deutsche Medicinische Wochenschrift*. No. 13. 1905.

9. Weissbart—"Styptol, a new hemostatic in gynecological practice." *Die Heilkunde*, October, 1904.

10. Freudenberg—"On Styptol." *Der Frauenzart*. No. 10. 1904.

"THE LAFAYETTE," 320 MANHATTAN AVE., NEW YORK.

